# Type 2 Diabetes: how informed are the general public? A cross-sectional study investigating disease awareness and barriers to communicating knowledge in high-risk populations in London

**DOI:** 10.1186/s12889-019-6460-7

**Published:** 2019-01-31

**Authors:** Reem Kayyali, Natasha Slater, Aisha Sahi, Deepa Mepani, Karima Lalji, Ako Abdallah

**Affiliations:** School of Life Sciences, Pharmacy and Chemistry, Kingston University London, Penrhyn Road, Kingston upon Thames, KT1 2EE UK

**Keywords:** Type 2 diabetes mellitus, Risk factors, Symptoms, Awareness

## Abstract

**Background:**

Preventing type 2 diabetes (T2DM) is one of the biggest health challenges currently facing the UK, with the NHS spending £14 billion each year on treating the disease and associated symptoms.

**Aims:**

The aim of this study was to determine the public’s awareness about the symptoms, risk factors and lifestyle choices, commonly associated with T2DM. This study also aimed to determine whether the level of awareness varies if the questions are asked in different languages, primarily those spoken by ethnic minorities.

**Methods:**

This was a cross sectional, multisite study conducted in London, UK, involving 399 participants, who were non-diabetic, aged between 25 and 74 years old and living in one of four selected London boroughs. Descriptive statistics, Chi square and Fisher’s Exact Tests were used to highlight and summarise the key findings of this study.

**Results:**

A response rate of 23.7% (*n* = 399/1683) for the English questionnaire was achieved. Overall, 59.4% (*n* = 237/399) of the cohort were able to identify a minimum of three T2DM symptoms and thus, were considered to have adequate or good awareness. Whereas, 60.6% (*n* = 242/399) were able to identify a minimum of six T2DM risk factors and were considered to have adequate or good awareness. More participants could correctly identify that obesity was a risk factor of T2DM when they were asked the question in their spoken language, rather than English (*p* < 0.01). When participants were asked about their current lifestyle choices, there were high levels of inactivity, smoking and alcohol consumption reported.

**Conclusion:**

Despite approximately half of participants demonstrating adequate or good awareness about the symptoms, risk factors and lifestyle choices commonly associated with T2DM, yet the study still highlights gaps in awareness among the remaining proportion of participants. Future prevention interventions should be tailored to address these existing gaps in awareness.

**Electronic supplementary material:**

The online version of this article (10.1186/s12889-019-6460-7) contains supplementary material, which is available to authorized users.

## Background

Over the past decade, the number of individuals with diabetes has risen by 59.8% in the United Kingdom (UK) and currently there are over 4 million individuals living with the condition. By 2025, this number is anticipated to rise to 5 million [1]. At present, diabetes care is costing the National Health Service (NHS) £14 billion per year and one factor contributing to the rising NHS costs is the late recognition of diabetes symptoms [2]. Consequently, UK healthcare providers are facing mounting pressures to address the issue; however, this is a challenging task for all healthcare providers, particularly those located in London, as the city is demographically and socio-economically diverse.

Diabetes is a condition characterised by either an absence of, or resistance to insulin, an endogenous protein that is responsible primarily for controlling blood sugar levels. Type 1 diabetes mellitus is prevalent in younger populations with an inability to produce insulin from the pancreas, the organ responsible for insulin secretion and blood sugar regulation. Type 2 diabetes mellitus (T2DM) refers to the latter mechanism whereby despite insulin production, the body’s response is diminished, and adequate control of blood sugar is not achieved. Without appropriate diagnosis and treatment, diabetes increases the risk of serious complications such as coronary heart disease and stroke.

This study was conducted in four London boroughs. According to national statistics, 40% of individuals residing in London are categorised as high risk for developing T2DM because of their ethnicity [3]. In a recent publication, it was reported that individuals of South Asian or African Caribbean origin are twice as likely to develop the disease compared to Caucasian individuals [4]. In addition, the prevalence of T2DM is increasing among adolescents and paediatric individuals, especially among those who belong to high risk ethnic groups [5]. This issue is particularly problematic in London as there is a younger population compared to other parts of the UK [3]. There is a high proportion of individuals who are living in London, who are not proficient in English and it is likely that these individuals will communicate in one of the 104 other languages spoken within the city [6].

Health literacy and language prove to be determinant factors for particular groups of patients at higher risk of T2DM and associated cardiovascular diseases. A cross-sectional study examining awareness of T2DM and heart disease among South Asian men and women living in the UK found that 28% (*n* = 92) did not understand the term diabetes, with 20% (*n* = 64) unable to suggest a single preventative measure [7]. This finding was also apparent among patients who had already received a diagnosis of diabetes. Significant variation in diabetes knowledge associated with ethnicity was observed with those demonstrating less awareness being Asian and Afro-Caribbean participants in comparison to their Caucasian counterparts in the study [8].

There are varying socio-economics throughout London and research has shown that individuals living in lower socioeconomic areas are 77% more likely to develop T2DM compared to individuals living in higher socioeconomic areas [9]. The four boroughs selected for this study have the highest levels of poverty within London [3]. Previous research has also shown that physical activity, successful smoking cessation and the consumption of fresh fruit and vegetables is lowest among individuals who reside in lower socioeconomic areas [10].

Inappropriate decisions about dietary choices and physical activity are contributory factors to the rising levels of obesity and thus, an obese individual is up to 80 times more likely to develop T2DM, compared to an individual who maintains a healthy weight [2].

To tackle the rising prevalence of T2DM, several large-scale studies have been undertaken to determine whether interventions, such as group exercise classes, nutritional counselling or the use of mobile apps to encourage weight loss can prevent T2DM [11, 12]. One study [13] reported that individualised dietary plans and weekly circuit training sessions over a one-year period, successfully delayed or prevented the onset of T2DM among the intervention group. Whilst, another prevention study concluded that lifestyle interventions were as effective as pharmacological interventions in reducing the incidence of T2DM [14]. Although T2DM prevention strategies have been widely researched, most prevention strategies have focussed on lifestyle modifications, rather than aiming to raise the public’s awareness about T2DM symptoms and risk factors. Despite previous research identifying disparity in knowledge of T2DM amongst high-risk ethnicities in the UK, little work has been done since the last decade to further investigate this relationship [15].

Therefore, the primary aim of the study was to investigate the current awareness of T2DM amongst adults living in Boroughs in London with high reported incidence of T2DM. The first objective was to investigate awareness in relation to risk-factors and symptoms of T2DM. The second objective, was to approach high-risk groups of participants from particular ethnic minorities at an increased risk of T2DM, to examine the extent of which language may be a barrier to communication of knowledge. The third objective was to examine participant lifestyle to identify potential relationships between patient behaviour and knowledge.

## Methods

This is a quantitative study involving a survey of adults living in four London boroughs (Brent, Ealing, Harrow and Newham) where there is a high prevalence of T2DM.

Ethical approval was sought and obtained from the Faculty of Science, Engineering and Computing Ethics Committee at Kingston University (ref 1213/045).

### Participants and recruitment

Members of the public were selected using convenience sampling. Approximately 1.17 million individuals reside within the four selected London boroughs [16]; therefore, to ensure this research is representative of the populations living within the selected boroughs, a power calculation was carried out. The calculation of sample size was based on the Raosoft sample size calculator using a 5% margin of error and at a 95% confidence interval [http://www.raosoft.com/samplesize.html]. For this study it was recommended to use a minimum sample size of 385 participants based on the total population of adults aged 25–74 across the boroughs where recruitment for the study took place [16].

To meet the inclusion criteria, participants must be aged between 25 and 74 years, have no previous diagnosis of diabetes and must live in one of the selected London boroughs. Recent literature has recommended a broader age range be applied to adolescence in the UK from 10 to 24 years of age [17]. This study aimed to examine awareness in adults and therefore the lower age limit of 25 years was set to reflect current recommendations. NHS health checks target those aged 40–74 and therefore an appropriate upper age limit of 74 years was set for the study. Participants were approached in person by the four researchers (AS, DM, KL, AA) in shopping centres, train stations and bus stops during a two-week period between February 2016 and March 2016.

Participants were provided with an information sheet to read that accompanied the questionnaire. Completion of the questionnaire signified implied consent on the part of the participants. The information sheet outlined the research aims and objectives and provided information regarding the questionnaire content, confidentiality, the right to participate or withdraw, as well as the contact details for the research team. Participants completed the questionnaires with the researchers administering the questions following provision of the information sheet. Initially, all documents were printed in English and the completed questionnaires were collected immediately with most participants completing the questionnaire in 15–20 min. On a separate occasion, the researcher based in Ealing revisited the borough and asked eligible participants who could communicate in Hindi, Punjabi or Urdu to complete the same questionnaire in their preferred language. Similarly to the recruitment phase in the other boroughs, participants were approached in shopping centres, bus stops and the train station in Ealing to complete the questionnaire. All questionnaires were collected immediately after completion, by the researcher. None of the questionnaires were incomplete, hence all questionnaires were considered for data analysis.

### Questionnaire

Data collection was performed using a paper-based questionnaire which consisted of 54 questions (see Additional file 1). The questions were predominantly closed ended or multiple-choice questions. Participants were asked questions to assess their awareness about T2DM symptoms and risk factors, using questions which had been adapted from validated screening tools including The Starr County Diabetes Education Study [18] and the Diabetes UK “Know your risk” tool [19]. Participants were also asked about their smoking habits, diet, physical activity levels. To determine participant’s current alcohol consumption, questions from AUDIT-C were used [20]. Several validated pictorial questions were also utilised to assess the publics’ perceptions of different body mass index (BMI) classifications [21]. The demographics section was at the end of the questionnaire. Knowledge level was assessed based on classification from other validated tools for assessing knowledge in other long-term conditions with categorisation into the following levels: < 50% = poor, 50–75% = adequate, ≥75 = good [22].

### Pilot study

After ethical approval, a pilot study was conducted involving 40 participants (10 from each borough) for face and content validation. Face validity included asking participants whether questions were clear and easy to complete. Content validity focused on completion of the questionnaire to determine whether suitable findings could be deduced from the questionnaire outcomes. Findings from the pilot study suggested that no changes were necessary. The individuals involved in the pilot study were excluded from the study to avoid any type of bias.

### Data analysis

Data was tabulated and analysed using Microsoft Excel. Descriptive statistics were used to determine particular key outcomes of study including: levels of awareness/knowledge with respect to symptoms and risk factors in T2DM, variation in knowledge based on lifestyle e.g. participant weight as well as variation in levels of awareness of T2DM across ethnicities. A Chi-Square Test was performed to evaluate differences in knowledge in relation to participants’ weight and physical activity. Additionally, a Fisher’s Exact Test was used to determine whether demographics or the language used to answer the questionnaire impacted individuals’ awareness about T2DM [23].

The level of statistical significance was set at *p* < 0.05.

## Results

### Response rate and demographics

A response rate of 23.7% (*n* = 399/1683) was achieved for the English questionnaire. This sample size meets the minimum sample size required by the power calculation. Charateristic details of the cohort are presented in Table 1.

**Table 1:** Sample characteristics of eligible study participants who completed English questionnaire

Parameter	Participant Data %(n)
Gender	
Male	44.4% (178)
Female	55.6% (221)
Age Range	
25–33 years	31.6% (126)
34–41 years	23% (92)
42–49 years	17% (68)
50–57 years	13.8% (55)
58–65 years	7.8% (31)
66–74 years	6.8% (27)
Ethnicity	
*White*	28.3%(113)
*Black*	15.3%(61)
*Asian*	40.9%(164)
*Other*	15.5%(62)
Main Spoken Language	
English	49.9%(200)
Urdu	9.5%(38)
Punjabi	9.1%(36)
Hindi	7.7%(31)
Other	23.8%(95)
Highest level of Education	
University	36.3%(146)
College	40.6%(162)/
Secondary School	19.5%(78)
Primary School	3.6%(14)
Number of participants who had received medical information about T2DM previously	40.6% (162)

### Symptoms awareness

Most participants, 68.4% (*n* = 273/399) and 65.2% (*n* = 260/399) respectively, could recognise that increased thirst and polyuria were both symptoms of T2DM; however, only 45.6% (*n* = 182/399), 48.9% (*n* = 195/399) and 57.6% (*n* = 230/399) of the cohort respectively could correctly identify that weight loss, blurred vision and lethargy were other common symptoms.

Overall, 59.4% (*n* = 237/399) of the participants could correctly identify at least three out of five common T2DM symptoms, with 91 patients (22.8%, *n* = 91/399) and 146 patients (36.6%, *n* = 146/399) demonstrating adequate and good levels of awareness of T2DM symptoms respectively. The results were stratified according to gender, age, ethnicity, highest level of education and exposure to medical information (Table 2). No statistically significant results were generated when the data was stratified according to age, ethnicity and educational level. However, more females had an adequate (*n* = 47/221) or good (*n* = 101/221) awareness of T2DM symptoms compared to males (*n* = 40/178 adequate awareness, (*n* = 49/178 good awareness), (*p* < 0.01). Poor level of awareness was more prominent in patients who had not received any medical information (*n* = 117/ 237) compared to those who had been exposed to medical information (*n* = 50/162) (*p* < 0.01) (Table 2).

**Table 2:** Statistical significance Table for Symptoms and Risk Factor awareness scores

	0–2 Symptoms (poor level)	3 Symptoms (adequate level)	4–5 Symptoms (good level)	*P* value	0–5 Risk factors (poor Level)	6–9 Risk factors (adequate level)	10–12 Risk factors (good level)	*P* value
Gender								
Male (n=)	89	40	49		83	77	17	
Female (n=)	73	47	101	< 0.01	73	118	31	0.01
Age								
24–49 years (n=)	106	62	114		102	141	39	
50–74 years (n=)	56	25	36	0.121	49	58	10	0.27
Ethnicity								
Lower-risk (n=)	50	24	44		41	59	18	
High-risk (n=)	103	56	91	0.90	108	114	28	0.24
Exposed to medical information								
Yes (n=)	50	38	74		52	82	28	
No (n=)	117	50	70	< 0.01	109	110	18	< 0.01
Highest Level of Education								
Primary or Secondary School (n=)	46	15	30		40	45	6	
College or University (n=)	120	70	118	0.129	117	151	40	0.20

### Risk factor awareness

**Fig. 1:**
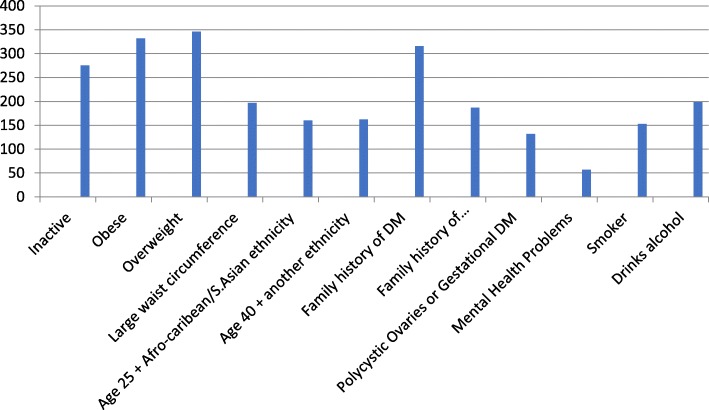
The number of participants who could correctly identify T2DM risk factors (*N* = 399)

Most of the cohort, 86.7% (*n* = 346/399) identified being overweight, and 83.2% (*n* = 332/399) identified obesity as potential risk factors that could increase the likelihood of a T2DM diagnosis, whereas fewer participants, 38.3% (*n* = 153/399) and 49.6% (*n* = 198/399) respectively, could identify that smoking and alcohol consumption were modifiable risk factors associated with T2DM. Only 14.3% (*n* = 57/399) of the cohort were aware that mental health conditions, such as depression and schizophrenia were risk factors for T2DM (Fig. 1). Overall, 60.6% (*n* = 242/399) of the cohort could correctly identify at least six out of twelve risk factors; with 11.8% (*n* = 47/399) having adequate awareness and 48.8% (*n* = 195/399) having good awareness of T2DM risk factors.

The results were stratified according to gender, age, ethnicity, highest level of education and exposure to medical information (Table 2). No statistically significant results were generated when the data was stratified according to age, ethnicity, level of education However, more females had an adequate (*n* = 118/222) or good (*n* = 31/222) awareness of T2DM risk factors compared to males (*n* = 77/177 adequate awareness, *n* = 17/177 good awareness) (*p* < 0.01).

Again, poor level of awareness about T2DM risk factors was more prominent in patients who had not received any medical information (*n* = 109/ 237) compared to those who had been exposed to medical information (*n* = 52/162) (p < 0.01) (Table 2).

### Lifestyle choices

Most of the cohort, 74.7% (*n* = 298/399) reported that they exercised on a regular basis. Walking, gardening and housework were defined as non-vigorous activities in the questionnaire; whereas running, cycling, swimming and weightlifting were defined as vigorous activities. Only 41.9% (*n* = 125/298) of those who exercised, reported that their activities were vigorous. Only 15.1% (*n* = 45/298) of the participants were meeting the current exercise recommendations outlined by the Department of Health [24].

A Chi-Square test was performed to examine potential differences in participants’ awareness based on physical activity. The results indicated a significant difference (*p* = 0.01) in awareness of risk factors between participants indicating high level of physical activity compared to those with low level of physical activity; the classification of physical activity level was based on the recommendations outlined by the Department of Health [24].

Participants were asked about their smoking history and current alcohol consumption. Almost half of the cohort, 42.3% (*n* = 169/399) reported that they either currently smoked or had smoked previously, with the modal number of cigarettes smoked per day being 10–19. In terms of alcohol consumption, 50.6% (*n* = 202/399) of the cohort reported that they regularly consumed alcohol, with one quarter of the cohort 25.1% (*n* = 100/399) reporting that they drank at least twice a week.

Participants were also asked to recall information about their diet during a typical week. With regards to snacking, most participants, 87.5% (*n* = 349/399), reported that they snacked between meals and 45.5% (*n* = 159/349) opted for crisps, chocolate bars or biscuits as their preferred snack. Fewer participants, 15.1% (*n* = 53/349) opted for fruit or nuts. Participants were also asked about their consumption of desserts. Most participants, 81.0% (*n* = 323/399), reported eating at least one dessert per week; however, 19.2% (*n* = 62/323) reported eating a minimum of five desserts each week. Furthermore, when asked about their sugary drink consumption, 64.1% (*n* = 256/399) reported drinking a minimum of one sugary drink per day.

To determine participants awareness of dietary requirements and calorie consumption, they were asked “What is the recommended daily calorie intake for men and women?”. Overall, 27.3% (*n* = 109/399) were aware that a typical male should consume 2500 kcal per day whereas 34.1% (*n* = 136/399) were aware that a typical female should consume 2000 kcal per day. Participants that answered incorrectly were more likely to overestimate the recommended daily calorie intake rather than underestimate it.

### Body mass index (BMI) recognition

Using a validated pictorial tool, participants were shown images of overweight and obese individuals [17]. Few participants, 7.8% (*n* = 31/399) could identify the images representing overweight individuals; whereas, most participants, 77.4% (*n* = 309/399), could identify the images representing the extreme scale of obesity (BMI > 40 kg/m2; Class III). None of the participants could identify the early stages of obesity (BMI 30–34.9 kg/m2, obesity class I) as obese, but few 23% (*n* = 92/399) identified them as being overweight (BMI 25–29.9 kg/m2).

Participants were asked to provide their weight, height and BMI measurements. Most participants, 89.7% (*n* = 358/399), were unable to calculate their BMI whilst only 58.6% (*n* = 234/399) knew their height and slightly more, 62.5% (*n* = 250/399), knew their current weight.

Additionally, a Chi-Square test was performed to examine potential differences in participants’ awareness based on their weight. The calculation compared participants with a normal weight (BMI = 18.5–24.9) to those who were considered overweight or obese (BMI ≥25). There was no statistically significant variation in awareness (*p* = 0.43) between the weight categories.

### General awareness

Participants were asked 20 subsequent questions about T2DM which were adapted from the Starr County Diabetes Education Study [15] and the DiabetesUK “Know your risk” tool (Table 3) [19]. It was assumed that individuals who could correctly answer 10 out of the 20 questions had an adequate or good level of awareness about T2DM.

**Table 3:** The number of participants who correctly answered the general knowledge questions

Question	No of correct responses n (%) (*n* = 399)
T_2_DM is a curable condition	208 (52.1%)
T_2_DM is caused by eating lots of sugary based foods	72 (18.1%)
Wound healing is delayed in people with T_2_DM	169 (42.4%)
People with T_2_DM are more likely to develop kidney problems	212 (53.1%)
Diabetes can increase the risk of heart attacks and other heart conditions	213 (53.4%)
The only medicine available for T_2_DM is insulin injection	174 (43.6%)
People with diabetes should exercise regularly	288 (72.2%)
T_2_DM can increase the risk of Alzheimer’s disease	52 (13.0%)
Sudden weight gain is a symptom of T_2_DM	113 (28.3%)
Feeling hungry often is a symptom of T_2_DM	155 (38.9%)
Women can get diabetes during pregnancy	229 (57.4%)
As a complication, diabetes can cause decay of limbs leading to amputation	186 (46.6%)
Diabetes can cause eye complications such as glaucoma	211 (52.9%)
Diabetes can cause loss of sensation in hands, legs and feet	160 (40.1%)
Diabetes is contagious	326 (81.7%)
Diabetes can make you gain more muscle mass	169 (42.4%)
In untreated diabetes, the amount of sugar in the blood usually increases	253 (63.4%)
Shaking and sweating are signs of low blood sugar levels	194 (48.6%)
Alcohol can increase the risk of diabetes due to high sugar content	254 (63.7%)
Insulin is produced in the kidneys	132 (33.1%)

The results were stratified according to gender, age, ethnicity, highest level of education and exposure to medical information (Table 4). No statistically significant results were detected when the results were stratified according to ethnicity; however more females (*n* = 113/221) had an adequate awareness about T2DM, compared to males (*n* = 59/178, *p* < 0.01). In addition, more participants aged between 24 and 29 years (*n* = 134/286) had an adequate or good awareness about T2DM, compared to participants who were aged between 50 and 74 years (*n* = 38/113, *p* = 0.01). Also, more individuals who had studied at college or university (*n* = 149/308) had an adequate or good awareness about T2DM, compared to individuals who reported their highest level of education to be primary or secondary school (*n* = 25/91, *p* < 0.01).

**Table 4:** Statistical Significance Table for general awareness scores

	0–9 Correct answers (poor awareness)	10–20 Correct answers (adequate or good awareness)	*P* value
Gender			
Male (n=)	117	59	
Female (n=)	110	113	< 0.01
Age			
24–49 years (n=)	151	134	
50–74 years (n=)	76	38	0.01
Ethnicity			
Low-risk (n=)	75	45	
High-risk (n=)	126	110	0.11
Exposed to medical information			
Yes (n=)	71	88	
No (n=)	156	84	< 0.01
Highest Level of Education			
Primary or Secondary School (n=)	65	25	
College or University (n=)	160	149	< 0.01

Finally, more individuals who had been exposed to medical information about T2DM (*n* = 88/159) had an adequate or good awareness about T2DM, compared to individuals who had not received any previous information (*n* = 84/240, *p* < 0.01).

### Medical information

Participants were asked several further questions about T2DM information. Less than half of the cohort, 40.6% (*n* = 162/399), had received information about T2DM previously. Of those who had received information, 48.7% (*n* = 79/162) reported that the information was provided in a leaflet format; however, 60.5% (*n* = 98/162) would prefer to receive this information from healthcare professionals (HCPs).

### Language

For the second part of this study, individuals living in Ealing were approached and asked to complete the questionnaire in Hindi, Punjabi or Urdu, depending upon their main spoken language. Variable response rates were achieved. The Hindi questionnaire had a response rate of 49.2% (*n* = 31/63) whereas the Urdu and Punjabi questionnaires had similar response rates of 43.7% (n = 31/71) and 43.2% (*n* = 32/74) respectively. Demographic details of the cohort are presented in Table 5.

**Table 5:** Demographics of eligible study participants who completed questionnaire in Hindi, Punjabi and Urdu

Parameter	Participant Data
Gender	
Male	55% (*n* = 52)
Female	45% (*n* = 42)
Modal Age	42–49 years
Asian Ethnicity	100%
Indian	29% (*n* = 27)
Pakistani	71% (*n* = 67)
Highest level of Education	
University	19.4%(*n* = 18)
College	48.0% (*n* = 45)
Secondary School	30.6% (*n* = 29)
Primary School	2.0% (*n* = 2)

Throughout this study, the same questionnaire was utilised and participants were awarded one point for each correctly identified symptom, risk factor or general information about T2DM, thus the maximum score that could be obtained was 37. The mean scores were calculated and stratified according to language. Ealing participants who completed the Hindi, Urdu and English questionnaire achieved mean score of 15, 16 and 17 respectively whereas the participants who completed the Punjabi questionnaire achieved the highest mean score of 18 out of 37. There were no statistically significant differences between mean scores for each language.

The questionnaire results were further analysed and it was observed that participant awareness about obesity as a risk factor of T2DM changed when asked in different languages. More participants could correctly identify that obesity was a risk factor for T2DM when asked in Punjabi, Hindi or Urdu, compared to when they were asked in English (*p* < 0.01). However, there was no statistically significant difference observed with other options/statements listed as risk factors or to assess general awareness in the survey when participants were asked in English or their main spoken language.

## Discussion

By 2025, there will be 5 million individual living with diabetes in England [3]. To tackle this growing epidemic, several large-scale studies have investigated whether intensive lifestyle interventions can successfully prevent or delay a T2DM diagnosis [11, 12]. Most interventions have focussed on an individual’s nutritional status or their current level of physical activity, rather than raising awareness about T2DM symptoms and risk factors [13, 14]. To address the existing gap in the literature regarding awareness about T2DM symptoms and risk factors among the public, this study investigated the public’s awareness about T2DM symptoms and risk factors, in addition to determining whether communication of knowledge was impacted by potential language barriers among high-risk ethnicities.

Although the study was conducted across boroughs with high T2DM prevalence, more than half of the participants were deemed to have an adequate or good level of awareness about the condition, irrespective of whether they were questioned in English or their main spoken language. This finding is somewhat indicative of a shift in awareness, with previous evidence suggesting inconsistency in knowledge of T2DM between ethnic groups, with those most at risk demonstrating the least awareness [7, 8, 15]. .In contrast, this study has identified equivalence of awareness of T2DM across ethnicities, suggesting that within a modern multi-cultural urban setting such as London, language is less of a barrier to knowledge communication. However, despite this finding, prevalence of poor knowledge was still evident across the rest of the participant population irrespective of ethnicity.

Most participants recognised that excessive thirst or polyuria were symptoms of T2DM; however, few could recognise other common symptoms. Similarly, most participants recognised that being overweight or obese increased the likelihood of a T2DM diagnosis; however, significantly less participants could recognise other modifiable risk factors, such as smoking or excessive alcohol consumption. In some cases, T2DM may be asymptomatic; however, if high-risk individuals are aware of the symptoms and risk factors, it may result in disease prevention, or an early diagnosis and a reduced likelihood of complications, such as retinopathy and neuropathy [25].

Discrepancies between patient reported awareness and behaviour were observed during the study particularly with respect to physical activity. The current exercise guidelines from the Department of Health recommend that individuals should participate in 75 min of vigorous activity or 150 min of non-vigorous activity each week [24]. However, despite 68.9% (*n* = 275/399) of participants identifying not exercising as a risk factor for T2DM, only 15.1% (*n* = 45/298) of those who exercised met the guideline recommendations. However, those who met the exercise recommendations had higher level of awareness of T2DM risk factors (*p* < 0.01). Additionally, 55.4% (*n* = 221/399) of the cohort reported between 0 and 20 min of activity per week. This finding is concordant with a national US survey that identified that high levels of awareness of the role of exercise in reducing the risk for T2DM does not necessarily correlate with increased exercise engagement in both high-risk and diagnosed diabetic patients [26]. Nevertheless our findings suggest that although knowledge of risks does not enhance exercise engagement, it enhances the proportion of those engaged in exercising in meeting the exercise recommendations.

Stratification of results according to participant demographics revealed no statistically significant associations between age, ethnicity, educational level and T2DM awareness. However, females and those who had received information about T2DM previously had a greater awareness of T2DM symptoms and risk factors. The latter finding is supported by results from a meta-analysis which concluded that lifestyle education could reduce the 1-year incidence of T2DM by 50%, thus emphasizing the importance of providing information for T2DM prevention [27].

Of those who had received information about T2DM previously, most reported that it had been supplied in a leaflet format. However, 60.5% of the cohort would prefer to receive this information verbally from a HCP. A systematic review which determined the effectiveness and value of written information among patients with long term conditions, reached a similar conclusion [28]. The review revealed that when patients were provided with written information, they did not value it. In addition, the patients who were happy to receive written information emphasised that it should not be used as a substitute for spoken information provided by an HCP.

Findings from this study revealed that T2DM awareness was not influenced by language. This may have been due to the small sample size used in the second part of this study. Although the findings were not statistically significant, it is important to consider spoken languages, particularly in multi-cultural cities, like London. Offering medical information in different languages ensures that key health messages are distributed throughout the population.

This study has several strengths and limitations. Firstly, it was conducted prior to the launch of the NHS Diabetes Prevention Programme (DPP) [29]. Eventually, the NHS DPP will be rolled out across the four London boroughs visited during this study.

Thus, this study provides an insight into the current levels of awareness about T2DM and may provide a helpful baseline for the NHS DPP. Once the programme has finished, this study could be repeated to determine whether T2DM awareness has improved.

This study had an acceptable sample size across the 4 included boroughs; however, the convenience sampling methods used to recruit participants may have been a limitation, as the researchers were only able to recruit participants in public places; therefore, home-bound individuals or those who did not use public transport were not accurately represented. Compared to London population, the sample size of the study can be considered small (given the low response rate) which might limit the generalisability of the results across all boroughs in London. Also, participants were asked to self-report information about their lifestyles. This method of data collection relies on an individual being able to accurately recall information and thus, could be influenced by cognitive decline or memory recall issues [30]. The participant sampling method was chosen based on previous evidence of higher response rates in face-to-face surveys compared to postal surveys [31]. Furthermore, this study targeted members of the public hence access to postal addresses was not feasible as this would constitute a breach of confidentiality. However, this approach of on the spot completion in addition to the length of the questionnaire, may have impacted the response rate with only 23.7% (*n* = 399/1683) of those asked to take part completing the questionnaire. Finally, the data collected was quantitative based on options provided in the data collection tool regarding symptoms and risks awareness, hence the results would have benefited from further qualitative research to explore trends of awareness in more details.

## Conclusions

More than half of the participants in this study demonstrated adequate or good knowledge and awareness about the symptoms, risk factors and lifestyle choices, commonly associated with T2DM. However, some gaps in awareness still exist among the remaining proportion of participants. The existing evidence suggests that T2DM prevention interventions can be beneficial; however, future prevention strategies must be tailored according to the needs of the local population, and should aim to raise awareness about the condition. Given the role of awareness in disease prevention, it would be valuable to investigate public awareness of other long-term conditions as well. Finally, this study showed that T2DM awareness was not influenced by language; however, further research involving a larger cohort is required.

## Additional file

Additional file 1: FINAL Project diabetes questionnaire – BMC. (DOCX 251 kb)

## Abbreviations

BMI: Body Mass Index
DPP: Diabetes Prevention Programme
HCP: Healthcare Professional
NHS: National Health Service
T2DM: Type 2 Diabetes Mellitus
