# Supplementary material for: Type 2 Diabetes: how informed are the general public? A cross-sectional study investigating disease awareness and barriers to communicating knowledge in high-risk populations in London
Source: BMC Public Health. 2019 Jan 31;19:138. doi: 10.1186/s12889-019-6460-7 (PMC6357431; doi:10.1186/s12889-019-6460-7)
Supplement: Supplementary file 1 — FINAL Project diabetes questionnaire – BMC. (DOCX 251 kb) [file 12889_2019_6460_MOESM1_ESM.docx]

**Diabetes Knowledge Questionnaire**

**Please tick the option you think is correct.**

1. **Which of the following do you think is a symptom of diabetes?**
2. **A person is more likely to get Type 2 diabetes if he/she**

|  | Yes | No | Don’t know |
| --- | --- | --- | --- |
| Feeling thirsty |  |  |  |
| Urinating more frequently |  |  |  |
| Weight loss |  |  |  |
| Lack of energy |  |  |  |
| Blurred vision |  |  |  |

|  | Yes | No | Don’t know |
| --- | --- | --- | --- |
| Doesn’t exercise |  |  |  |
| Is overweight |  |  |  |
| Is obese |  |  |  |
| Has a large waist circumference |  |  |  |
| Is over the age of 25 years old and from an African-Caribbean, Black African or South Asian ethnicity |  |  |  |
| Is over the age of 40 years old and from another ethnic background |  |  |  |
| Has a family history of diabetes |  |  |  |
| Has a history of high blood pressure, heart attack or stroke |  |  |  |
| Has a history of polycystic ovaries, gestational diabetes |  |  |  |
| Has History of schizophrenia, bipolar disorder or depression |  |  |  |
| Is a smoker |  |  |  |
| Drinks alcohol |  |  |  |

**This is one unit of alcohol…**

**…and each of these is more than one unit**

**Please tick the option you think is correct.**

1. **How often do you have an alcoholic drink?**

🞏 Never (Skip to question 7) 🞏 Once a month or less 🞏 2-4 times per month

🞏 2-3 times per week 🞏 4+ times per week 🞏 Not sure

1. **What is your alcohol intake on a typical day when you are drinking?**

🞏 1-2 units 🞏 3-4 units 🞏 5-6 units 🞏 7-9 units 🞏 10+ units

1. **How often do you consume 6 or more (women) or 8 or more (men) alcohol units on one occasion?**

🞏 Never 🞏 Less than monthly 🞏 Monthly 🞏 Weekly 🞏 Daily or almost daily

1. **Rate the following alcoholic beverages, in terms of sugar content, from 1-5 in the boxes below (1 being the lowest sugar content and 5 being the highest)**


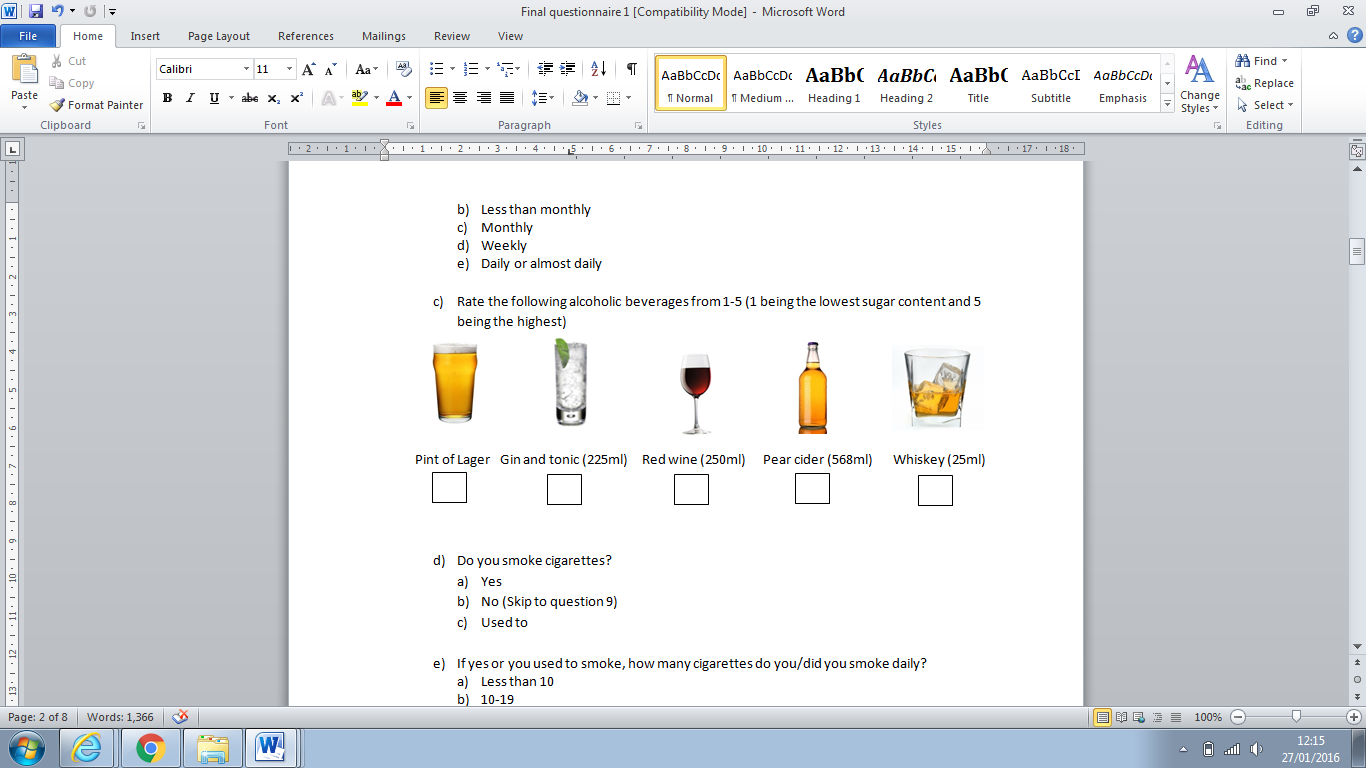


1. **Do you smoke cigarettes?**

🞏 Yes 🞏 No (Skip to question 9) 🞏 Used to

1. **If yes or you used to smoke, how many cigarettes do you/did you smoke daily?**

🞏 Less than 10 🞏 10-19 🞏 20-29 🞏 30-39 🞏 40+

1. **What type of physical activity do you do in a typical week? (Tick all that apply)**

🞏 None (Skip to question 11) 🞏 Walking 🞏 Cycling 🞏 Running 🞏 Swimming 🞏 Gardening 🞏 Housework (eg. Vacuuming. DIY)

🞏 Other (please specify)……………………………………………………….

1. **How many minutes of physical activity do you do in a typical week?**

🞏 Under 20 🞏 21-60 🞏 61-90 🞏 91-120 🞏 121-149 🞏150+

1. **What is the recommended daily calorie intake for men?**

🞏 1000 🞏 1500 🞏 2000 🞏 2500 🞏 3000 🞏3500

🞏 Not sure

1. **What is the recommended daily calorie intake for women?**

🞏 1000 🞏 1500 🞏 2000 🞏 2500 🞏 3000 🞏3500

🞏 Not sure

1. **In a typical week how often do you have a dessert (e.g. cake, custard, ice-cream, biscuits)?**

🞏 Never 🞏1-2 🞏 3-4 🞏 5-6 🞏 7 or more

1. **On a typical day how often do you have a snack between meals?**

🞏 Never (Skip to question 16) 🞏 1 🞏 2 🞏 3 🞏 4 🞏 4 +

1. **What would be a typical snack for you? (Tick all that apply)**

🞏 Fruit 🞏 Crisps 🞏 Chocolate Bar 🞏 Nuts 🞏Cereal Bar

🞏Biscuits 🞏Other (please specify)………………………………………………..

1. **In a typical day how many non-diet beverages do you drink? (squash, fizzy drink, juice etc**)

🞏 Never 🞏 Less than 2 🞏 2-5 🞏 More than 5

**Please tick the following statements depending on whether you think they are true or false. If you are not sure, please tick the ‘Don’t Know’ option.**

|  | Questions | True | False | Don’t Know |
| --- | --- | --- | --- | --- |
| 17 | Type 2 Diabetes is a curable condition |  |  |  |
| 18 | Type 2 Diabetes is caused by eating lots of sugar based foods |  |  |  |
| 19 | Wound healing is delayed in people with type 2 diabetes |  |  |  |
| 20 | People with Type 2 diabetes are more likely to develop kidney problems |  |  |  |
| 21 | Diabetes can increase the risk of heart attacks and other heart conditions |  |  |  |
| 22 | The only medicine available for Type 2 diabetes is insulin injection |  |  |  |
| 23 | Diabetics should exercise regularly |  |  |  |
| 24 | Type 2 Diabetes can increase the risk of Alzeihmer’s disease. |  |  |  |
| 25 | Sudden weight gain is a symptom of Type 2 diabetes |  |  |  |
| 26 | Feeling hungry often is a symptom of Type 2 diabetes |  |  |  |
| 27 | Women can get diabetes during pregnancy |  |  |  |
| 28 | As a complication, diabetes can cause decay of limbs leading to amputation |  |  |  |
| 29 | Diabetes can cause eye complications such as glaucoma |  |  |  |
| 30 | Diabetes can cause loss of sensation in hands, legs and feet |  |  |  |
| 31 | Diabetes is contagious |  |  |  |
| 32 | Diabetes can make you gain more muscle mass |  |  |  |
| 33 | In untreated diabetes, the amount of sugar in the blood usually increases |  |  |  |
| 34 | Shaking and sweating are signs of low blood sugar levels |  |  |  |
| 35 | Alcohol can increase the risk of diabetes due to high sugar content |  |  |  |
| 36 | Insulin is produced in the kidneys |  |  |  |

**37.** **Have you ever been exposed to medical information about Type 2 diabetes?**

🞏 Yes 🞏 No (skip to question 40)

**38. How was this medical information presented to you?**

🞏 Television 🞏 Leaflets 🞏 Billboards 🞏 Radio

🞏 Newspaper 🞏 Poster 🞏 Other (please specify) …………………………

**39. Which method would you prefer to receive medical information about Type 2 diabetes?**

🞏 Doctor Surgery 🞏 Pharmacy 🞏 Television 🞏Leaflets 🞏Billboards

🞏 Newspaper 🞏 Poster 🞏 Other (please specify)…………………………………………

**40. Do you know your weight?**

🞏 Yes (please specify)……………………… 🞏 No

**41. Do you know your height?**

🞏 Yes (please specify)……………………… 🞏 No

**42. Do you know your BMI (body mass index)?**

🞏 Yes (please specify)………………………. 🞏 No (skip to question 44)

**43. What is the classification of your BMI (body mass index)?**

🞏 Don’t Know 🞏 Underweight 🞏Normal Weight 🞏 Overweight 🞏 Obese

**44. Which of the images in your opinion show an overweight person? (Tick more than one if applicable)**

🞏 A 🞏 B 🞏 C 🞏 D 🞏 E

🞏 F 🞏 G 🞏 H 🞏 I 🞏 J

**45. Which of the images in your opinion show an obese person? (Tick more than one if applicable)**

🞏 A 🞏 B 🞏 C 🞏 D 🞏 E

🞏 F 🞏 G 🞏 H 🞏 I 🞏 J

**46. What is your gender?**

🞏 Male 🞏 Female

**47. Which age group, in years, do you belong to?**

🞏 25-33 🞏 34-41 🞏 42-49 🞏 50-57 🞏 58-65 🞏 66-74

**48. Which ethnic group do you belong to?**

🞏 White British 🞏 White Other 🞏 Indian/ British Indian 🞏 Pakistani/ British Pakistani

🞏 Arab/ British Arab 🞏 Black African/ British Black African

🞏 Black Caribbean/ British Black Caribbean 🞏 Other (please specify)…………………………………...

**49. What is the main language you converse in on a daily basis?**

🞏 English 🞏 Hindi 🞏 Urdu 🞏 Punjabi 🞏 Polish 🞏 Arabic 🞏 Somali 🞏 Other (please specify)……………………………

**50. Has a friend or family member been diagnosed with Type 2 Diabetes?**

🞏 Yes 🞏 No 🞏 Don’t Know

**51. How often do you need help reading/ understanding medical information?**

🞏 Rarely 🞏 Never 🞏 Often

**52. Do you work or study in a healthcare related field?**

🞏 Yes 🞏 No

**53. What is the highest level of education you have completed?**

🞏 None 🞏Primary School 🞏 Secondary School 🞏College 🞏University or above

**54. What is you annual income?**

🞏 Below £10,000 🞏 £10,000-20,000 🞏 £20,000-30,000 🞏 £30,000-40,000

🞏 £40,000-50,000 🞏 £50,000-60,000 🞏 £60,000-70,000 🞏 £70,000 or above

🞏 Prefer not to say

**Thank you for your participation**
